# Achievements and Challenges of Social Epidemiology Research Aiming to Reduce Health Inequality: A Revised English Version of Japanese in the Journal of the Japan Medical Association 2020;149(9):1626-30

**DOI:** 10.31662/jmaj.2021-0176

**Published:** 2021-12-28

**Authors:** Katsunori Kondo

**Affiliations:** 1 Department of Social Preventive Medical Sciences, Center for Preventive Medical Sciences, Chiba University, Chiba, Japan; 2 Department of Gerontological Evaluation, Center for Gerontology and Social Science, National Center for Geriatrics and Gerontology, Obu, Japan

**Keywords:** Health inequalities, Social epidemiology, Social determinants of health, Social capital

## Abstract

Health inequalities are defined as “gaps in health status between groups, which are created by differences in community or socioeconomic status.” In response to the General Assembly Resolution (2009) of the World Health Organization, the World Medical Association issued a statement in the same year, and the Japanese health policy “Health Japan 21 (second term)” indicated a “reduction of health inequalities” as the basic direction. In 2000, we described the presence of health inequalities in Japan, which was regarded as a relatively egalitarian country. This was the starting point of the Japan Gerontological Evaluation Study. It was developed into large longitudinal studies that reveal the significance of “social determinants of health” that cause health inequalities. We verified the feasibility and effects of healthy aging policies by fostering social capital through community intervention studies. These findings and knowledge have been translated into municipal and central government policies. Here we review what has been achieved and the remaining challenges in more than 20 years of social epidemiological research.

## Introduction

Health inequalities are defined as “gaps in health status between the groups, which are created by differences in community or socioeconomic status” ^(2), (3)^. In response to the General Assembly Resolution (2009) ^(4)^ of the World Health Organization (WHO), the World Medical Association issued a statement ^(5)^ in the same year, and the Japanese health policy “Health Japan 21 (second term: 2013-2023)” ^(2)^ indicated a “reduction of health inequalities” as the basic direction. The author has been working on the study of health inequalities since 1999. In 2020, he received an award from the Japanese Medical Association for his study on social epidemiology aimed at reducing health inequalities ^(1)^. In this paper, we describe the process and review the achievements over the past 20 years and the remaining challenges.

## Inspiration: from Clinical Experience to Being a Research Fellow in the United Kingdom

When I was in my 30s, I was a registered specialist of rehabilitation medicine in the treatment of stroke patients. When analyzing the databases of our patients, I found that 4.8% of them received public assistance, which was an impediment to home discharge ^(6)^. I hypothesized that poverty might be a risk factor for strokes because, at that time, 1.7% of the individuals over 70 years old received public assistance nationally, which accounted for one-third of our patients. Then, I realized the importance of social determinants in health and medical care and concluded that “socioeconomic factors should be taken into consideration” ^(5)^.

After moving to Nihon Fukushi University, I asked a municipality to provide data on the taxable income and certification status of the eligibility for the benefit of long-term care insurance. After analyzing the relationship between those, I found that the prevalence of the certification rate, which reflects the prevalence of those with disabilities, was about five times higher in the low-income group relative to the high-income group ^(7)^.

Fortunately, I had an opportunity to visit the United Kingdom as a research fellow at the University of Kent at Canterbury from 2000 to 2001. When I studied in the UK, I found that “health inequalities” were one of the main themes at the international congress of health economics, and Prime Minister Blair established a committee in 1997, which attracted attention in terms of policy as well. The book, entitled “Social Epidemiology,” was published in 2000 and defined social epidemiology as “the branch of epidemiology that studies the social distribution and determinants of health” ^(8)^. I also came across Wilkinson’s book ^(9)^, in which he argued that Japan was an egalitarian country with rich social cohesion and that this may contribute to longevity. Social cohesion, also known as social capital, is defined as “the resources that are accessed by individuals as a result of their membership of a network or a group” ^(10)^. Social epidemiology researchers hypothesized that societies with smaller inequalities had relatively rich social capital levels compared to those with large inequalities, and people living in egalitarian societies with rich social capital levels have better health statuses ^(8), (9)^. Verification of these hypotheses was in progress.

In Japan, growing socioeconomic inequality became a hot issue during the early 2000s. Therefore, if the hypotheses were right, the widening of such inequalities would have been a threat in Japan as well. Consequently, as a researcher in social medicine and social policy, I thought it would be worthwhile to work on reducing health inequalities through social epidemiological research.

## From Returning to Japan to Publishing the “Health Gap Society”

In 2003, in cooperation with 15 municipalities predominantly in Aichi Prefecture, 32,891 independent older individuals without certification of the eligibility for the benefit of long-term care insurance were surveyed. We confirmed consistent health inequalities between municipalities or communities and different socioeconomic groups. A higher prevalence of older people with declining cognitive and oral functions, with malnutrition, and who were homebound (going out less than once per week), as well as poor social capital-related indicators (such as less participation in community organizations and less social support), were observed among those with lower educational attainment or lower income. For example, it was found that the prevalence of depression in men measured on the Geriatric Depression Scale (GDS-15 item version, ≥10) was 2.3% within the highest income group (the equivalent income was 4 million yen or more per year) and 15.8% within the lowest income group (less than 1 million yen per year). The largest health inequality was reached approximately seven times ^(11), (12), (13)^.

We thought such findings of health inequalities within the Japanese society should be published and responded to by the society. This is because increases were observed in the number of unstable/non-regular employed individuals, single people, income disparities, and relative poverty rates in the 2000s. Therefore, I published the “Health Gap Society-What Undermines Mind and Health” ^(12)^, in 2005, in which the mechanisms that cause health inequalities and the social policies that can serve as countermeasures were reviewed. This book, which emphasizes the importance of bio-psycho-social models and social policy for the population’s health, won an award from the Japan Association for Social Policy Studies. The work was an “early warning,” which is one of the roles of epidemiology.

## Launch and Development of the Japanese Gerontological Evaluation Study (JAGES)

Various questions on health inequalities should be researched. Are they reproducible in other municipalities and communities at different times? Are they also observed in objective measures, such as mortality and certification of eligibility for the benefit of long-term care insurance, which is regarded as a functional decline? Are they not “reverse causalities” or “apparent” associations that are often observed in cross-sectional analyses? What are the mechanisms that cause health inequalities? What countermeasures are possible? Are they effective?

First, the results of the large-scale survey in 2003 were published in Japanese ^(11)^ and English ^(13)^. Large-scale surveys were repeated in 2006, 2010, 2013, 2016, and 2019, and an increasing number of municipalities (insurers of public long-term care insurance) cooperated. Since 2010, when the municipalities who cooperated increased to 12 prefectures across Japan, the name was changed from the Aichi Gerontological Evaluation Study (AGES) to JAGES ^(14), (15)^. In 2019, about 250,000 older individuals from 64 municipalities of 25 out of 47 prefectures responded nationwide. A large-scale repeated cross-sectional and longitudinal database for social epidemiology research has been established, including data on approximately 750,000 older people) ^(15)^.

We provided feedback to municipalities on the results of the analysis to contribute to “Evidence-Based Policy Making” or “Knowledge Translation” ^(15)^ to develop business plans for long-term care insurance and long-term care prevention. We also made efforts to build a trusting relationship with municipalities, and municipalities providing data of long-term care insurance gradually increased. In addition, the panel survey data was compiled from the responses of the same population three years later. These data enabled us to conduct longitudinal studies to verify the “cause of cause” ^(12), (15), (16)^.

By creating a system for repeated surveys every three years in multiple municipalities, it enables research with a natural experimental design. In Iwanuma City, Miyagi Prefecture, which was affected by the Great East Japan Earthquake and Tsunami in 2011, we surveyed all older people seven months before the disaster by chance. Because the opportunity to conduct a natural experiment is very rare, we could collaborate with Professor Kawachi at the Harvard T.H. Chan School of Public Health and obtain grants from the National Institute of Health of the USA to the Iwanuma project ^(17), (18), (19)^.

These databases enabled the publication of over 600 papers (over 250 papers in English) ^(20)^. Data are available regardless of affiliation if the conditions are met. From 2018 to 2019, there were over 61 affiliated institutions among the lead authors, which indicated that many researchers have utilized these databases ^(15)^.

## Ecological Study: Is It Reproducible?

**Figure 1.**
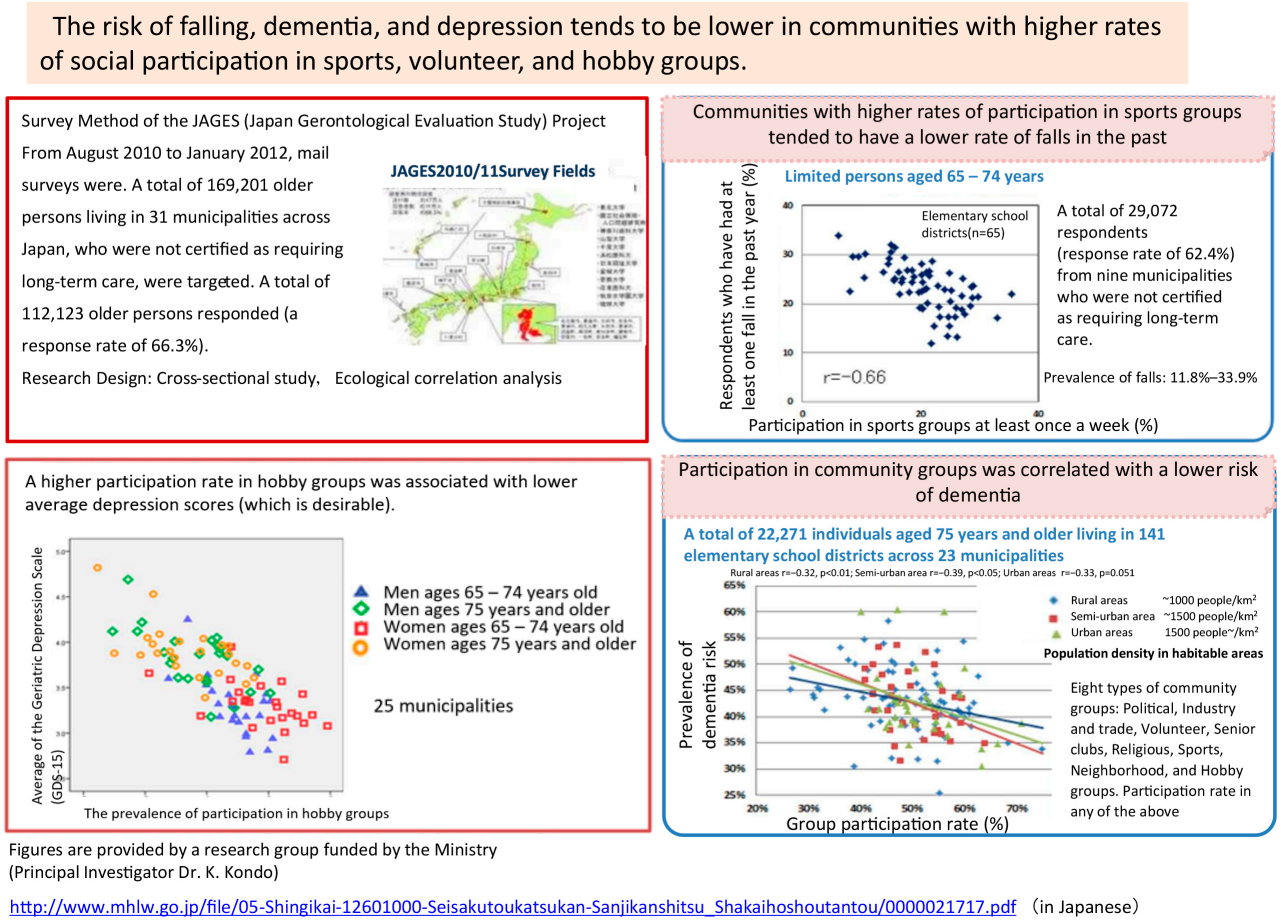
Social Participation and Long-Term Care Prevention.

Using data derived from all years, we confirmed reproducibility of significant health inequalities between municipalities and communities within municipalities. Next, using cross-sectional ecological analyses, we found correlations between social capital-related indicators represented by health status and, for example, the percentage of older people who participated in community groups ^(14)^. For example, the historical prevalence of falls indicates three-fold differences, from 11.8% to 33.9%, between the elementary school areas. The prevalence was higher in areas with lower participation rates in sports groups (Figure 1, upper right). The mean scores on the Geriatric Depression Scale (lower scores indicate better mental health) were poor where participation rates in hobby groups were low (lower left). The risk of dementia was also high in areas where participation in any of the eight community groups was low (lower right). This data was presented at the National Council of Social Security of the Ministry of Health, Labour, and Welfare in 2013 and served as an evidence basis for one of the reviews of the long-term care prevention policy in Japan.

## Elimination of “Reverse Causality” by Longitudinal Studies.

It is important to note, however, that “reverse causality” cannot be ruled out in the cross-sectional analysis, as shown in Figure 1. Participation in sports groups does not inhibit falls; on the contrary, individuals can participate in sports associations because they have not fallen. On the other hand, a lower income may not compromise health; conversely, their incomes may remain low because they were unhealthy. Eliminating reverse causality requires longitudinal studies that are restricted to healthy individuals only, examine temporally preceding participation status or income, and identify subsequent deterioration in health status.

The longitudinal study confirmed that the risk of requiring long-term care, rate of certification for long-term care benefits, and mortality rate were low in both communities and individuals with higher participation rate or frequency in sports and hobbies. For example, in a four-year longitudinal study, the hazard ratio (HR) was 0.83, 0.72, and 0.57 for participation in one, two, and three or more types of organizations compared to those that did not participate in any organizations ^(21)^. It has also been shown that low-income individuals tend to be certified as needing long-term care or die ^(3), (22)^.

## Clarifying the Causal Process of Health Inequalities

**Figure 2.**
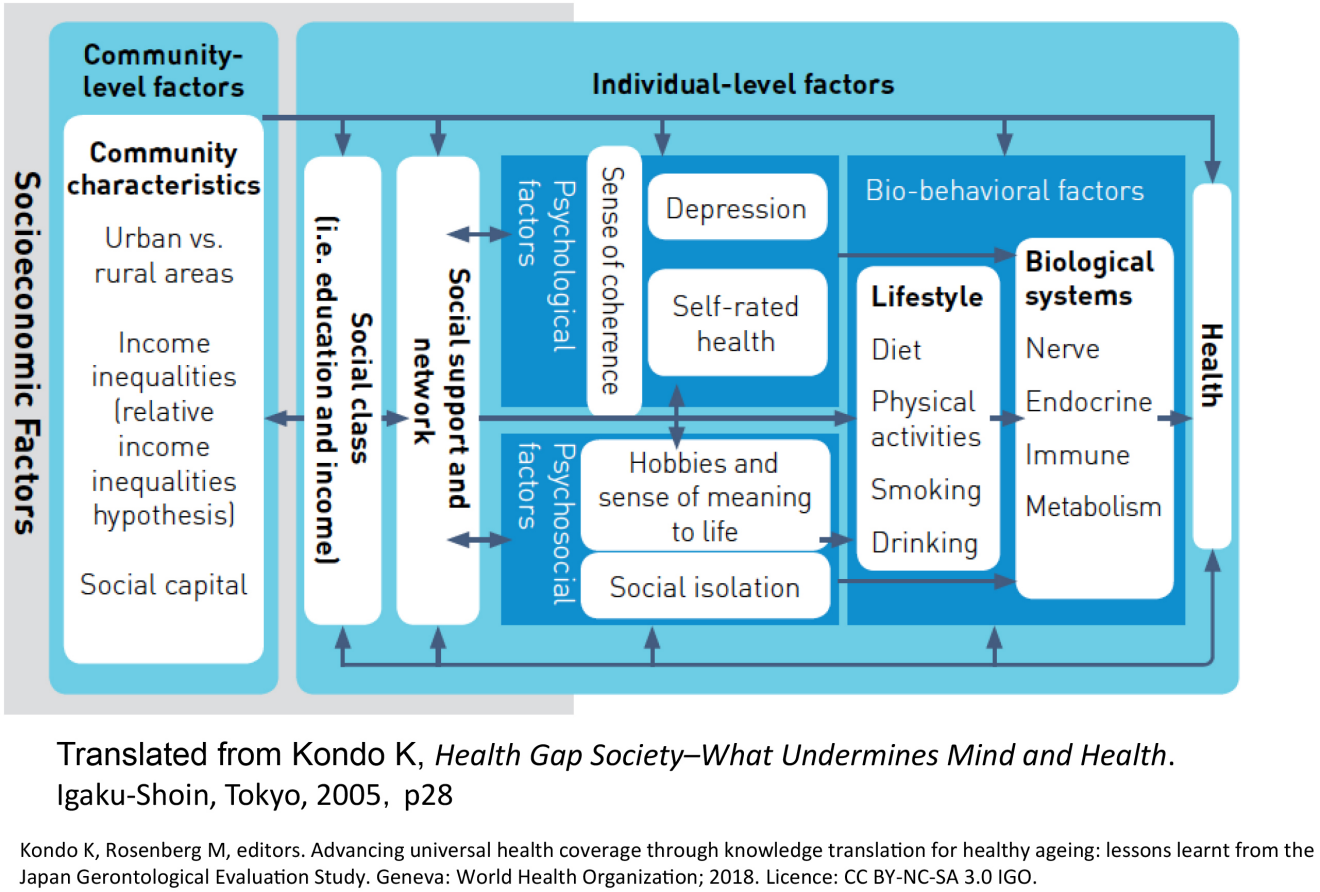
Conceptual Framework of the Social Determinants of Health.

The process of producing health inequalities is complex (Figure 2) ^(12), (16), (23)^. First, it is affected by the municipal/community environment (on the left hand of Figure 2). Community settings include urban and rural areas, population density, the extent of social capital or income inequalities, and built environments ^(12), (16), (23), (24), (25), (26), (27), (28), (29), (30)^. For those in a disadvantaged position in the social hierarchy, external resources such as social support, which has a health protection effect, and the ability to survive (i.e., stress-coping ability), which is an internal resource, become scarce ^(12), (16), (23), (25), (31), (32)^. This situation causes stress reactions, such as depression, and leads to unhealthy behavior and an unhealthy physical state or cognitive decline resulting from the biological impact of stress ^(12), (16), (23), (31), (33)^.

Another aspect of the causal process of health inequalities is life course ^(3), (16), (34), (35), (36), (37), (38)^. For example, the probability of the novel onset of depressive symptoms in old age is 1.3 times higher in individuals who reported that their childhood was less privileged ^(39)^. Its impact throughout their life was also apparent―e.g., higher medical care costs in old age among individuals who were maltreated during childhood ^(40)^.

**Figure 3.**
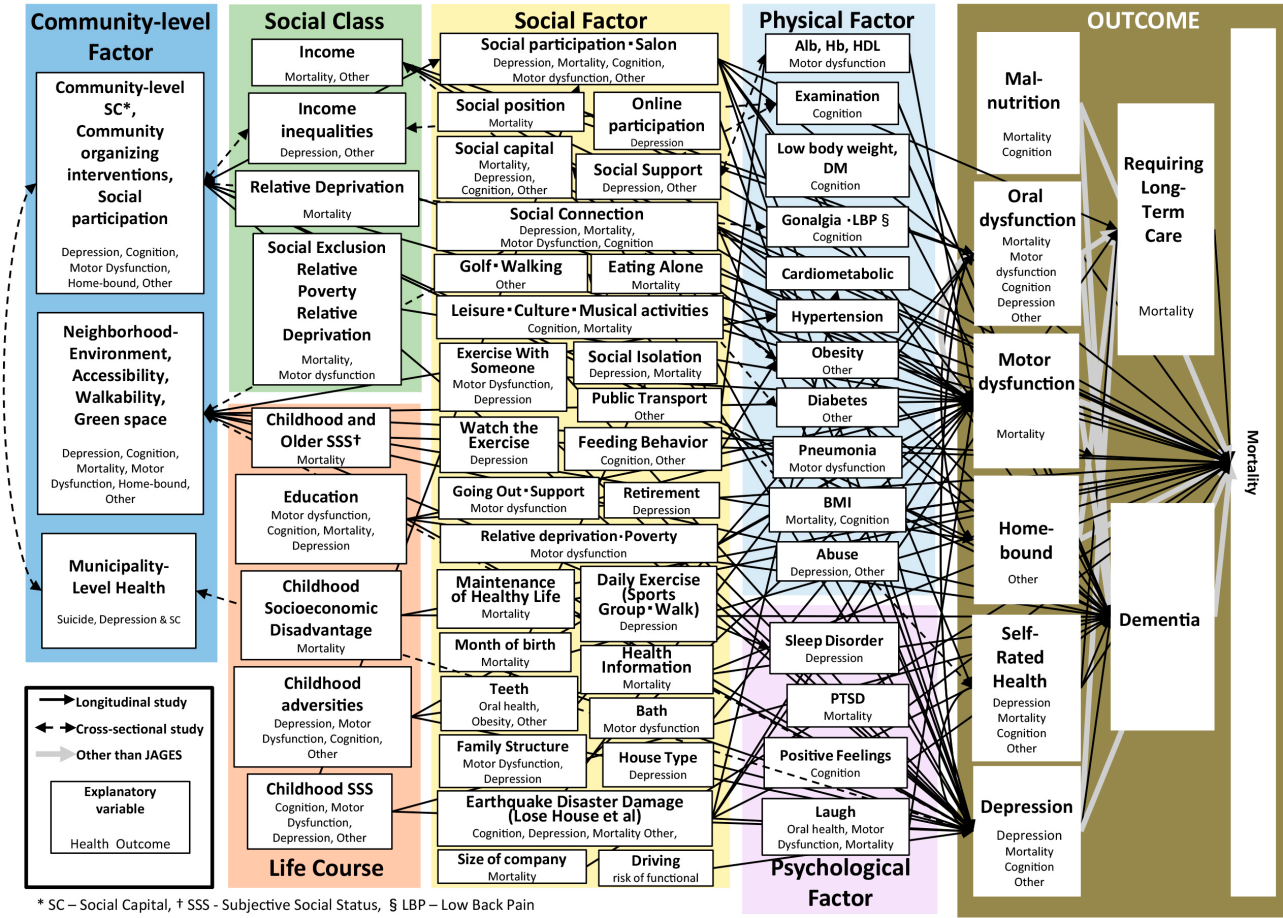
Determinants of Health Verified by JAGES.

Social determinants of health that have been analyzed and reported using the JAGES data are shown in Figure 3. In addition to cross-sectional studies, many longitudinal studies have confirmed the complex pathways presented in Figure 2 as theoretical hypotheses in 2005 ^(20)^. Bio-medical, psycho, social, and environmental factors determine the population’s health.

## Can We Increase Social Participation?

Can intended interventions increase social participation and improve health levels? Since 2006, we have also studied community interventions to verify this aspect. In Taketoyo Town, Aichi Prefecture, we established a “community salon” where older people can gather, enjoy, and interact, and we evaluated the process and the effects thereafter ^(15), (16), (41), (42)^.

As the number of salon sites, volunteers, and general participants increased, over 10% of the town’s older population began to participate. When followed up, 35.0%-87.5% of the participants reported increased social support ^(41)^. The salon became a place for health information exchange, and there was a ripple effect. Compared to non-salon participants, many participants reported participating in other sports and volunteer groups apart from the community salon ^(41)^.

When we compared the health conditions of participants and non-participants before and after the interventions, the health conditions before participation were better among participants ^(43)^. On the contrary, those who had a salon opened nearby were more likely to participate, and those for whom the salon was opened far away were less likely to participate ^(42)^. In other words, the distance from the salon to the participants’ residences was found to be an instrumental variable ^(42)^, which is a coin substitute in a randomized controlled trial. The analysis was conducted using this advanced analytical technique (instrumental variable methods), which is regarded as a pseudo-randomized controlled trial. Adjusting for the differences between the participating and non-participating groups, the participating group indicated that self-rated health improved by 2.5 times eight months after the beginning of the intervention ^(42), (43)^, and the incidence of certification for long-term care benefit over five years decreased by approximately half, i.e., 14.0% in the non-participating group vs. 7.7% in the participating group ^(44)^. Moreover, a comparison after seven years of follow-up showed that the risk of cognitive impairment among participants was also reduced by approximately 30% ^(45)^. The prevalence of participation was higher among those with lower incomes ^(46)^, so a reduction in health inequalities is expected.

## Knowledge Translation to Municipal and National Policies

In 2009, the WHO approved a resolution at the General Assembly to reduce health inequalities ^(4)^. Thereafter, the Monitoring Report Committee of the Japanese Society of Public Health and Science Council of Japan published recommendations in 2010-2011^(3)^. In the Health Japan 21 (Second term) ^(2), (3)^ published in 2012, the basic strategy was “to reduce health inequalities” by improving the quality of the social environment, such as increasing opportunities for social participation. In addition, the long-term care prevention policy against functional decline has been expanded from a high-risk approach to a population approach since the fiscal year of 2015 following the National Council of Social Security (Figure 1) ^(16)^. Municipalities, which provide public long-term care insurance, began to build communities that are easy to participate in.

One of the three recommendations of the WHO was the measurement and research of health inequalities ^(23)^. Thus, through a joint study with the WHO Centre for Health Development (the “WHO Kobe Centre”), which developed the Urban Health Equity Assessment and Response Tool (HEART) ^(47)^, we have developed a system for “visualizing” health inequalities and related factors ^(14), (15)^. The JAGES HEART ^(14)^ becomes a prototype of the Ministry of Health, Labour and Welfare’s “visualizing” system for community-based comprehensive care.

## Avenues for Future Research

We have pioneered the theory ^(12), (16)^ and empirical ^(3), (13), (16)^ and intervention studies ^(16), (41)^ on social epidemiology, and knowledge translations ^(15), (16)^ to a policy aimed at reducing health inequalities, which is a goal of both the WHO ^(4)^ and the Ministry of Health, Labour and Welfare, Japan ^(2)^. We described the actual state of health inequalities among older Japanese ^(3), (13), (16)^ and proposed the hypothesis of causal mechanisms (Figure 2) ^(12), (16)^; we verified that many psychosocial and environmental factors are involved in the causal process of health inequalities and clarified the importance of bio-psycho-social models (Figure 3). However, there are also many future research questions.

Further studies are required to elucidate the remaining mechanisms that impact health―from various environments spanning from built environments, life courses, and incentives to the internet―to find effective measures to reduce health inequalities. When considering measures, medicine based on the “bio-medical model” alone is not enough because of the complexity of the process of generating health inequalities and of the large impact of non-biological factors. The “prescription for the health gap society” ^(12)^ requires “health in all policies,” from measures for social isolation to educational policies and enhanced redistribution of incomes, as stated by the WHO ^(48)^. For example, what is needed for patients who suffer from depression with social isolation is not only antidepressants but also social prescriptions that connect depressed individuals with society. Evaluation of these interventions should include effectiveness, efficiency, and equity ^(16)^.

In the future, research toward the realization of a “primordial prevention” ^(49)^, which tackles “underlying conditions leading to causation” ^(49)^ and creates an environment in which one is healthy simply by living, is necessary.

## Article Information

This article is based on the study, which received the Medical Award of The Japan Medical Association in 2020. This is a revised English version of the article originally published in Japanese in the Journal of the Japan Medical Association 2020; 149(9) 1626-1630 ^(1)^. The original version is available at https://www.med.or.jp/cme/jjma/newmag/pdf/149091626.pdf.

### Conflicts of Interest

None

### Sources of Funding

JAGES (the Japan Gerontological Evaluation Study) was supported by MEXT (Ministry of Education, Culture, Sports, Science and Technology-Japan)-Supported the 21^st^ Century Center of Excellence (COE) Program (2003-2007) and the Strategic Research Foundation at Private Universities (2009-2013), Grant-in-Aid for Scientific Research (14310105, 18390200, 22330172, 22390400, 23243070, 23590786, 23790710, 24390469, 24530698, 24683018, 25253052, 25713027, 25870573, 25870881, 26285138, 26460828, 26780328, 26882010, 15H01972, 15H04781, 15H05059, 15K03417, 15K03982, 15K16181, 15K17232, 15K18174, 15K19241, 15K21266, 15KT0007, 15KT0097, 16H05556, 16K09122, 16K00913, 16K02025, 16K12964, 16K13443, 16K16295, 16K16595, 16K16633, 16K17256, 16K17281, 16K19247, 16K19267, 16K21461, 16K21465, 16KT0014, 17K04305, 17K04306, 18H03018, 18H04071, 18H03047, 18H00953, 18H00955, 18KK0057, 19H03901, 19H03915, 19H03860, 19K04785, 19K10641, 19K11657, 19K19818, 19K19455, 19K24060, 19K20909, 20H00557) from JSPS (Japan Society for the Promotion of Science); Health Labour Sciences Research Grants (H19-Choju-Ippan-027, H22-Choju-Shitei-008, H24-Junkanki [Seishu]-Ippan-007, H24-Chikyukibo-Ippan-009, H24-Choju-Wakate-009, H25-Kenki-Wakate-015, H25-Choju-Ippan-003, H26-Irryo-Shitei-003 [Fukkou], H26-Choju-Ippan-006, H27-Ninchisyou-Ippan-001 H28- Choju-Ippan-002, H28- Ninchisyou-Ippan-002, H30-Kenki-Ippan-006, H29-Chikyukibo-Ippan-001, H30-Jyunkankinado-Ippan-004, 19FA1012, 19FA2001, 21FA1012), Research project on health and welfare promotion for the elderly from the Ministry of Health, Labour and Welfare, Japan; the Research and Development Grants for Longevity Science from Japan Agency for Medical Research and development (AMED) (JP18dk0110027, JP18ls0110002, JP18le0110009, JP20dk0110034, JP21lk0310073, JP21dk0110037), the Research Funding for Longevity Sciences from National Center for Geriatrics and Gerontology (24-17, 24-23, 29-42, 30-30, 30-22, 20-19, 21-20); Open Innovation Platform with Enterprises, Research Institute and Academia (OPERA, JPMJOP1831) from the Japan Science and Technology (JST); a grant from the Japan Foundation For Aging And Health (J09KF00804); World Health Organization Center for Health Development (WHO Kobe Centre) [grant WHO APW 2017/713981]; a grant from Innovative Research Program on Suicide Countermeasures (1-4), a grant from Sasakawa Sports Foundation, a grant from Japan Health Promotion & Fitness Foundation, a grant from Chiba Foundation for Health Promotion & Disease Prevention, the 8020 Research Grant for fiscal 2019 from the 8020 Promotion Foundation (adopted number: 19-2-06), a grant from Niimi University (1915010), and grants from Meiji Yasuda Life Foundation of Health and Welfare.

### Acknowledgement

I would like to express my deepest gratitude for the funding received, the members of the Japan Gerontological Evaluation Study (JAGES), and everyone who supported our research.
